# Supplementary material for: Manipulating attention facilitates cooperation
Source: Commun Psychol. 2025 Mar 17;3:39. doi: 10.1038/s44271-025-00206-9 (PMC11913732; doi:10.1038/s44271-025-00206-9)
Supplement: Supplementary file 2 — Supplementary Materials [file 44271_2025_206_MOESM2_ESM.pdf]

SUPPLEMENTARY TABLES

Supplementary Table 1:

(a) Effects of payoffs and option positions on cooperation. Fixed effects coefficients' estimates of payoffs and option positions regressions on Cooperation using mixed-effects logistic models. All models include the normalized trial number as control variable. (1) Model including payoffs only. Normalized values for the Temptation, Reward, Punishment and Suckers payoffs are included. (2) Model including option positions only: C you right vs C you left and C other down vs C other up, where C stands for cooperation. (3) Model including the option positions and their interactions. (4) Model including both payoffs, option positions, and their interactions. All models include random intercepts and random slopes for each coefficient and each participant.

	**(1) Payoffs**	**(2) Positions**	**(3) Positions interactions**	**(4) Payoffs x Positions**
(Intercept)	-2.56***	-1.33***	-1.37***	-1.40***
	[-3.22; -1.90]	[-1.66; -1.01]	[-1.69; -1.04]	[-1.78; -1.03]
Temptation	-0.18*			0.02
	[-0.33; -0.03]			[-0.10; 0.13]
Reward	0.53***			0.18**
	[0.35; 0.72]			[0.06; 0.31]
Punishment	-0.76***			-0.46***
	[-0.92; -0.60]			[-0.57; -0.35]
Suckers	0.54***			0.35***
	[0.38; 0.69]			[0.25; 0.45]
Trial number	-0.30***	-0.12**	-0.12**	-0.12**
	[-0.47; -0.14]	[-0.20; -0.05]	[-0.20; -0.05]	[-0.20; -0.03]
Position columns (C right)		-0.03	0.03	0.03
		[-0.11; 0.05]	[-0.08; 0.14]	[-0.07; 0.14]
Position rows (C down)		0.16*	0.23**	0.26**
		[0.04; 0.29]	[0.08; 0.37]	[0.10; 0.41]
C you right x C other down			-0.12	-0.13*
			[-0.26; 0.02]	[-0.27; -0.00]
C you right x Reward				-0.09
				[-0.22; 0.04]
C other down x Reward				-0.13*
				[-0.26; -0.00]
C you right x Suckers				0.07
				[-0.06; 0.21]
C other down x Suckers				0.13
				[-0.01; 0.27]
C you right x Punishment				0.01
				[-0.08; 0.10]
C other down x Punishment				0.10
				[-0.02; 0.22]
C you right x Temptation				-0.02
				[-0.11; 0.06]
C other down x Temptation				-0.10*
				[-0.19; -0.01]

	(1) Payoffs	(2) Positions	(3) Positions interactions	(4) Payoffs x Positions
AIC	9868.72	10277.40	10280.66	9837.97
BIC	10077.51	10385.65	10435.31	11152.55
Log Likelihood	-4907.36	-5124.70	-5120.33	-4748.98
Num. obs.	16862	16862	16862	16862
Num. groups (participant ID)	88	88	88	88

*P<0.05. 95% confidence intervals in parentheses. The significant coefficients are shown in bold. AIC, Akaike information criterion; BIC, Bayesian information criterion.

(b) Replication: fMRI sample

	(1) Payoffs	(2) Positions	(3) Positions interactions
(Intercept)	-2.29***	-1.11***	-1.11***
	[-2.97; -1.61]	[-1.43; -0.79]	[-1.43; -0.79]
Temptation	-0.39***		
	[-0.53; -0.25]		
Reward	0.82***		
	[0.60; 1.03]		
Punishment	-1.19***		
	[-1.45; -0.92]		
Suckers	0.59***		
	[0.41; 0.76]		
Trial number	-0.35***	-0.16***	-0.16***
	[-0.50; -0.19]	[-0.22; -0.09]	[-0.23; -0.09]
Position columns (C right)		0.02	0.01
		[-0.05; 0.10]	[-0.10; 0.12]
Position rows (C down)		0.10*	0.09
		[0.01; 0.18]	[-0.03; 0.21]
C you right x C other down			0.03
			[-0.11; 0.17]
AIC	10024.64	11352.85	11354.17
BIC	10234.01	11461.42	11509.26
Log Likelihood	-4985.32	-5662.43	-5657.08
Num. obs.	17233	17233	17233
Num. groups (participant ID)	90	90	90

*P<0.05. 95% confidence interval in parentheses. The significant coefficients are shown in bold. AIC, Akaike information criterion; BIC, Bayesian information criterion.

Supplementary Table 2: Models of cooperative choices. Utility model equations of the different models of cooperative choices. All utility differences are then used to estimate probabilities of cooperating using a softmax function $P(Cooperate) = \frac{1}{1+e^{-\beta \Delta U}}$

**Model name**	**Utility equations**
Winning model	$U(Cooperate) = p \cdot R^{\rho} + (1-p) \cdot \left((1-\alpha)S^{\rho} + \alpha T^{\rho}\right)$<br>$U(Defect) = p \cdot ((1-\alpha)T^{\rho} + \alpha S^{\rho}) + (1-p) \cdot P^{\rho}$<br>$\Delta U = U(Cooperate) - U(Defect)$
Alternative model 1 (risk aversion transformation after implementing other-regarding preferences)	$U(Cooperate) = p \cdot R^{\rho} + (1-p) \cdot \left((1-\alpha)S + \alpha T\right)^{\rho},$<br>$U(Defect) = p \cdot \left((1-\alpha)T + \alpha S\right)^{\rho} + (1-p) \cdot P^{\rho},$<br>$\Delta U = U(Cooperate) - U(Defect)$
Alternative model 2 (constant bias b)	$U(Cooperate) = p \cdot R^{\rho} + (1-p) \cdot \left((1-\alpha)S + \alpha T\right)^{\rho},$<br>$U(Defect) = p \cdot \left((1-\alpha)T + \alpha S\right)^{\rho} + (1-p) \cdot P^{\rho},$<br>$\Delta U = U(Cooperate) - U(Defect) + b$
Alternative model 3 ($\alpha = 1$, fully selfish)	$U(Cooperate) = p \cdot R^{\rho} + (1-p) \cdot (\alpha T^{\rho})$<br>$U(Defect) = p \cdot (\alpha S^{\rho}) + (1-p) \cdot P^{\rho}$<br>$\Delta U = U(Cooperate) - U(Defect)$
Alternative model 4 ($\rho = 1$, risk neutral)	$U(Cooperate) = p \cdot R + (1-p) \cdot \left((1-\alpha)S + \alpha T\right)$<br>$U(Defect) = p \cdot ((1-\alpha)T + \alpha S) + (1-p) \cdot P$<br>$\Delta U = U(Cooperate) - U(Defect)$
Alternative model 5 ($\alpha = 1$, fully selfish, and constant bias b)	$U(Cooperate) = p \cdot R^{\rho} + (1-p) \cdot (\alpha T^{\rho})$<br>$U(Defect) = p \cdot (\alpha S^{\rho}) + (1-p) \cdot P^{\rho}$<br>$\Delta U = U(Cooperate) - U(Defect) + b$
Alternative model 6 (CARA risk aversion)	$f(x) = \frac{1-e^{-\rho x}}{\rho}$<br>$U(Cooperate) = p \cdot f(R) + (1-p)$<br>$\cdot \left((1-\alpha)f(S) + \alpha f(T)\right)$<br>$U(Defect) = p \cdot ((1-\alpha)f(T) + \alpha f(S)) + (1-p) \cdot f(P)$<br>$\Delta U = U(Cooperate) - U(Defect)$
Alternative model 7 (CRRA risk aversion)	$f(x) = \frac{x^{1-\rho}}{1-\rho}$<br>$U(Cooperate) = p \cdot f(R) + (1-p)$<br>$\cdot \left((1-\alpha)f(S) + \alpha f(T)\right)$<br>$U(Defect) = p \cdot ((1-\alpha)f(T) + \alpha f(S)) + (1-p) \cdot f(P)$<br>$\Delta U = U(Cooperate) - U(Defect)$
Alternative model 8 ($\rho = 1.1$, risk seeking)	$\rho = 1.1$<br>$U(Cooperate) = p \cdot R^{\rho} + (1-p) \cdot \left((1-\alpha)S^{\rho} + \alpha T^{\rho}\right)$<br>$U(Defect) = p \cdot ((1-\alpha)T^{\rho} + \alpha S^{\rho}) + (1-p) \cdot P^{\rho}$<br>$\Delta U = U(Cooperate) - U(Defect)$
Alternative model 9 (Charness-Rabin social preference, additional parameter μ)	$U(Cooperate) = p \cdot ((1-\alpha)R^{\rho} + \alpha(\mu R^{\rho} + 2(1-\mu)R^{\rho})$<br>$+ (1-p)$<br>$\cdot \left((1-\alpha)S^{\rho} + \alpha(\mu S^{\rho} + (1-\mu)(S^{\rho}\right.$<br>$\left. + T^{\rho})\right)$<br>$U(Defect) = p \cdot ((1-\alpha)T^{\rho} + \alpha(\mu T^{\rho} + (1-\mu)(T^{\rho} + S^{\rho})$<br>$+ (1-p)$<br>$\cdot \left((1-\alpha)P^{\rho} + \alpha(\mu P^{\rho} + 2(1-\mu)P^{\rho})\right)$<br>$\Delta U = U(Cooperate) - U(Defect)$
Alternative model 10 (p varies depending on R-P)	$U(Cooperate) = p_i \cdot R^{\rho} + (1-p_i) \cdot \left((1-\alpha)S^{\rho} + \alpha T^{\rho}\right)$<br>$U(Defect) = p_i \cdot ((1-\alpha)T^{\rho} + \alpha S^{\rho}) + (1-p_i) \cdot P^{\rho}$<br>$\Delta U = U(Cooperate) - U(Defect)$<br>$p_i = p_1 \; if \; R-P \geq 10$<br>$p_i = p_2 \; if \; R-P < 10$

Supplementary table 3: Model comparison. Gray rows indicate weak difference between the models' goodness-of-fit.

Model name	AIC	BIC	ΔBIC
Winning model	141.9	154.9	0
Alternative model 1 (risk aversion transformation after implementing other-regarding preferences)	144.4	157.4	2.477
Alternative model 2 (constant bias b*)*	143	159.3	4.351
Alternative model 3 ($\alpha = 1$*, fully selfish)*	179.5	189.3	34.34
Alternative model 4 ($\rho = 1$*, risk neutral)*	145.4	155.1	0.2055
Alternative model 5 ($\alpha = 1$*, fully selfish, and constant bias* b*)*	147.3	160.3	5.4
Alternative model 6 (CARA risk aversion)	144.4	157.4	2.487
Alternative model 7 (CRRA risk aversion)	163.2	176.2	21.25
Alternative model 8 ($\rho = 1.1$*, risk seeking)*	145.5	155.2	0.3202
Alternative model 9 (Charness-Rabin social preference, additional parameter μ*)*	145.7	161.9	7.009
Alternative model 10 (p varies depending on R-P)[1]	144.5	160.8	5.84

[1]While we found a small increase in p in trials with a higher R—P difference (0.47 vs 0.43 across all participants), this increase was not statistically significant (t(65) = 0.65, p = 0.52).

Supplementary Table 4: Effects of relative sampling times on cooperation. Fixed effects coefficients' estimates of relative sampling time for the different AOIs (time spent looking at each AOI divided by trial duration) on Cooperation using mixed-effects logistic models. Trial number is not included as a control variable due to convergence issues.

	**Relative sampling time**		
(Intercept)	-1.41***	AIC	8264.68
	[-1.79; -1.02]	BIC	9068.90
T you	-2.34***	Log Likelihood	-4028.34
	[-2.93; -1.75]	Num. obs.	16862
T other	2.96***	Num. groups: subj_nb	88
	[2.19; 3.72]		
R you	2.15***		
	[1.54; 2.75]		
R other	5.20***		
	[4.37; 6.02]		
P you	-3.65***		
	[-4.27; -3.04]		
P other	-0.50		
	[-1.30; 0.29]		
S you	0.70*		
	[0.09; 1.31]		
S other	0.88*		
	[0.11; 1.65]		
Temptation payoff	-0.11**		
	[-0.17; -0.04]		
Reward payoff	0.26***		
	[0.17; 0.35]		
Punishment payoff	-0.32***		
	[-0.39; -0.24]		
Suckers payoff	0.25***		
	[0.18; 0.33]		

***P<0.001, **P<0.01, *P<0.05. 95% confidence interval in parentheses. The significant coefficients are shown in bold. AIC, Akaike information criterion; BIC, Bayesian information criterion.

Supplementary Table 5: Effects of option positions on fitted model parameters. Fixed effects coefficients' estimates of option positions regressions on model parameters using mixed-effects regression models. Only participants who cooperated or defected on more than one trial were used for the computational model fitting and therefore included in this analysis (N=66). (1) Effects of positions on Others regarding preference (α) parameter. (2) Model including interactions between row and columns positions. (3) Effects of positions on Risk aversion (ρ) parameter. (4) Model including interactions between row and columns positions. (5) Effects of positions on Belief (p) parameter. (6) Model including interactions between row and column positions. All models include random intercepts and random slopes for each coefficient and each participant.

	**(1) $\boldsymbol{\alpha}$**	**(2) $\boldsymbol{\alpha}$ inter**	**(3) $\boldsymbol{\rho}$**	**(4) $\boldsymbol{\rho}$ inter**	**(5) p**	**(6) p inter**
(Intercept)	0.75***	0.75***	0.92***	0.89***	0.43***	0.40***
	[0.69; 0.82]	[0.68; 0.82]	[0.76; 1.08]	[0.71; 1.06]	[0.36; 0.50]	[0.32; 0.48]
Position rows	-0.01	0.00	-0.01	0.05	0.11**	0.18**
(C down)	[-0.05; 0.03]	[-0.05; 0.05]	[-0.16; 0.13]	[-0.15; 0.26]	[0.03; 0.19]	[0.07; 0.29]
Position columns	-0.01	0.01	0.05	0.12	-0.02	0.05
(C right)	[-0.04; 0.02]	[-0.04; 0.05]	[-0.13; 0.23]	[-0.11; 0.35]	[-0.09; 0.06]	[-0.05; 0.16]
Position rows (C down) x Position columns (C right)		-0.03		-0.13		-0.14
		[-0.09; 0.03]		[-0.42; 0.16]		[-0.29; 0.01]
AIC	-88.34	-82.21	573.89	577.08	203.16	205.29
BIC	-52.58	-42.88	609.65	616.42	238.92	244.63
Log Likelihood	54.17	52.11	-276.94	-277.54	-91.58	-91.65
Num. obs.	264	264	264	264	264	264
Num. groups: subj	66	66	66	66	66	66

***P<0.001, **P<0.01, *P<0.05. 95% confidence interval in parentheses. The significant coefficients are shown in bold. AIC, Akaike information criterion; BIC, Bayesian information criterion.

Supplementary Table 6: Effects of the payoff value and fixation number (temporal order) on the share of trials spent looking at each location. Fixed effects coefficients' estimates of payoff and fixation numbers (temporal order, first, second or third fixation) regression on the share of trials spent looking at one screen location using mixed-effects regression. One model is fitted per location, using the proportion of trials in which the screen location is sampled at a given fixation: (1) Up left you, (2) Up left other, (3) Up right you, (4) Up right other, (5) Down left you, (6) Down left other, (7) Down right you and (8) Down right other.

	**(1) Up left you**	**(2) Up left other**	**(3) Up right you**	**(4) Up right other**	**(5) Down left you**	**(6) Down left other**	**(7) Down right you**	**(8) Down right other**
(Intercept)	1.19***	0.34***	1.07***	-0.78***	-0.31***	-0.32***	-0.30***	-0.87***
	[1.07; 1.32]	[0.22; 0.46]	[0.93; 1.20]	[-0.85; -0.71]	[-0.44; -0.18]	[-0.42; -0.22]	[-0.42; -0.17]	[-0.92; -0.81]
Fixation	-0.39***	-0.24***	-0.27***	0.11***	0.26***	0.08***	0.30***	0.15***
	[-0.44; -0.34]	[-0.28; -0.21]	[-0.31; -0.22]	[0.09; 0.13]	[0.21; 0.30]	[0.05; 0.11]	[0.25; 0.34]	[0.13; 0.16]
Payoff	0.44***	0.22***	0.41***	0.09***	0.41***	0.17***	0.31***	0.07***
	[0.40; 0.48]	[0.19; 0.24]	[0.37; 0.45]	[0.07; 0.10]	[0.37; 0.44]	[0.14; 0.19]	[0.28; 0.35]	[0.06; 0.09]
AIC	6698.45	5110.40	6832.87	2310.10	6392.95	4703.45	6411.61	2148.22
BIC	6727.31	5139.27	6861.74	2338.97	6421.82	4732.31	6440.47	2177.08
Log Likelihood	-3344.22	-2550.20	-3411.44	-1150.05	-3191.48	-2346.72	-3200.80	-1069.11
Num. obs.	2376	2376	2376	2376	2376	2376	2376	2376
Num. groups: subj	88	88	88	88	88	88	88	88

***P<0.001, **P<0.01, *P<0.05. 95% confidence interval in parentheses. The significant coefficients are shown in bold. AIC, Akaike information criterion; BIC, Bayesian information criterion.

Supplementary Table 7: Effects of AOI locations on frequency of sampling of the AOI during the first fixation. Fixed effects coefficients' estimates of positions of the AOI on the screen regressed on the share of trials for which the AOI is sampled first using mixed-effects regression. One model is fitted per AOI, using the proportion of trials on which the AOI is looked at on fixation 1 as the dependent variable: (1) Temptation you, (2) Temptation other, (3) Reward you, (4) Reward other, (5) Punishment you, (6) Punishment other, (7) Suckers you and (8) Sucker's other (9) Temptation other including interactions between row and column positions.

	**(1)** $\mathbf{T_Y}$	**(2)** $\mathbf{T_O}$	**(3)** $\mathbf{R_Y}$	**(4)** $\mathbf{R_O}$	**(5)** $\mathbf{P_Y}$	**(6)** $\mathbf{P_O}$	**(7)** $\mathbf{S_Y}$	**(8)** $\mathbf{S_O}$	**(9)** $\mathbf{T_O}$ **inter**
(Intercept)	0.14***	0.00	0.14***	0.00	0.09***	-0.00	0.06***	-0.00	0.03**
	[0.11; 0.17]	[-0.02; 0.02]	[0.12; 0.17]	[-0.01; 0.02]	[0.07; 0.12]	[-0.01; 0.01]	[0.04; 0.07]	[-0.01; 0.01]	[0.01; 0.05]
AOI up	0.16***	0.06***	0.15***	0.04***	0.18***	0.03*	0.10***	0.02**	0.01
	[0.12; 0.19]	[0.03; 0.09]	[0.12; 0.19]	[0.02; 0.06]	[0.14; 0.22]	[0.01; 0.05]	[0.08; 0.12]	[0.00; 0.03]	[-0.02; 0.04]
AOI left	0.05**	0.15***	-0.04*	0.12***	-0.01	0.10***	-0.03**	0.05***	0.09***
	[0.01; 0.09]	[0.12; 0.17]	[-0.08; -0.00]	[0.10; 0.13]	[-0.03; 0.02]	[0.08; 0.11]	[-0.05; -0.01]	[0.04; 0.06]	[0.07; 0.12]
AOI up x AOI left									0.10***
									[0.07; 0.14]
AIC	-281.22	-517.84	-366.66	-701.03	-377.43	-743.93	-702.21	-1196.24	-543.41
BIC	-242.58	-479.20	-328.03	-662.39	-338.79	-705.29	-663.57	-1157.60	-500.91
Log Likelihood	150.61	268.92	193.33	360.51	198.72	381.96	361.10	608.12	282.70
Num. obs.	352	352	352	352	352	352	352	352	352
Num. groups: subj	88	88	88	88	88	88	88	88	88

***P<0.001, **P<0.01, *P<0.05. 95% confidence interval in parentheses. The coefficients of interest are represented in bold. AIC, Akaike information criterion; BIC, Bayesian information criterion.

Supplementary Table 8: Effects of looking at each AOI on fixation 1, 2, or last on cooperation. Fixed effects coefficients' estimates of dummies representing fixations of different AOI at different time points regressed on Cooperation using mixed-effects logistic models. Dummies for the first, second, and last fixation were included as well as trial number as a control variable.

	**Fixations dummies**		**Fixations dummies (continued)**
(Intercept)	-3.87***	P you first fixation	0.79
	[-4.85; -2.89]		[-0.23; 1.81]
T you first fixation	1.39**	P other first fixation	1.41**
	[0.44; 2.33]		[0.40; 2.42]
T other first fixation	1.33**	P you second fixation	0.37
	[0.33; 2.34]		[-0.08; 0.81]
T you second fixation	0.67**	P other second fixation	0.97***
	[0.22; 1.12]		[0.46; 1.49]
T other second fixation	1.22***	P you last fixation	-1.63***
	[0.71; 1.73]		[-2.35; -0.91]
T you last fixation	-1.24***	P other last fixation	-1.09**
	[-1.96; -0.51]		[-1.86; -0.33]
T other last fixation	-0.06	S you first fixation	1.47**
	[-0.80; 0.69]		[0.47; 2.47]
R you first fixation	1.37**	S other first fixation	1.38**
	[0.41; 2.33]		[0.33; 2.43]
R other first fixation	1.59**	S you second fixation	0.94***
	[0.62; 2.56]		[0.46; 1.42]
R you second fixation	0.77***	S other second fixation	0.90**
	[0.34; 1.19]		[0.30; 1.50]
R other second fixation	1.72***	S you last fixation	-0.60
	[1.26; 2.17]		[-1.30; 0.11]
R you last fixation	-0.12	S other last fixation	-0.56
	[-0.81; 0.58]		[-1.39; 0.27]
R other last fixation	0.70	Trial number	-0.27***
	[-0.01; 1.40]		[-0.43; -0.11]
AIC	9935.76		
BIC	12851.03		
Log Likelihood	-4590.88		
Num. obs.	16862		
Num. groups: subj_nb	88		

***P<0.001, **P<0.01, *P<0.05. 95% confidence interval in parentheses. The significant coefficients are shown in bold. AIC, Akaike information criterion; BIC, Bayesian information criterion.

Supplementary Table 9: Effects of looking at each AOI on fixation 1, 2, or last on cooperation for the four options positions. Fixed effects coefficients' estimates of dummies representing fixations of different AOI at different time points regressions on Cooperation using mixed-effects logistic models. Dummies for the first, second, and last fixation were included. We could not include the trial number as a control variable in this analysis due the models not converging when adding this variable. One regression is fitted for each option position (1) Cooperate up left, (2) Cooperate up right, (3) Cooperate down left, (4) Cooperate down right.

	**(1)**<br>**CC up left**	**(2)**<br>**CC up right**	**(3)**<br>**CC down left**	**(4)**<br>**CC down right**
(Intercept)	-4.84***	-10.75*	-6.14***	-6.57**
	[-6.23; -3.45]	[-20.81; -0.69]	[-9.26; -3.02]	[-10.78; -2.36]
T you first fixation	1.04	3.24	2.97	4.06
	[-0.63; 2.70]	[-3.44; 9.93]	[-0.03; 5.96]	[-0.30; 8.43]
T other first fixation	1.66	0.74	3.21*	3.31
	[-0.04; 3.35]	[-6.52; 8.00]	[0.22; 6.21]	[-1.27; 7.90]
T you second fixation	0.78	1.13*	0.84	1.07
	[-0.16; 1.72]	[0.17; 2.10]	[-0.44; 2.13]	[-0.33; 2.47]
T other second fixation	1.67***	1.20	2.22**	1.54
	[0.78; 2.55]	[-0.09; 2.49]	[0.75; 3.68]	[-0.04; 3.13]
T you last fixation	-1.22	3.81	-1.36	-2.06**
	[-2.67; 0.24]	[-4.12; 11.75]	[-3.58; 0.87]	[-3.62; -0.50]
T other last fixation	-0.30	5.56	0.41	-0.02
	[-1.87; 1.28]	[-2.39; 13.52]	[-1.88; 2.69]	[-1.56; 1.52]
R you first fixation	1.45	2.45	3.46*	3.90
	[-0.19; 3.10]	[-4.33; 9.22]	[0.48; 6.43]	[-0.45; 8.25]
R other first fixation	1.50	2.79	3.01	4.31
	[-0.16; 3.16]	[-4.28; 9.85]	[-0.05; 6.07]	[-0.46; 9.09]
R you second fixation	1.29**	0.44	0.98	0.66
	[0.45; 2.13]	[-0.54; 1.43]	[-0.34; 2.30]	[-0.74; 2.07]
R other second fixation	2.15***	1.37	2.34***	2.07*
	[1.29; 3.02]	[-0.23; 2.97]	[1.07; 3.61]	[0.47; 3.67]
R you last fixation	-0.37	5.23	-0.03	-0.41
	[-1.86; 1.11]	[-2.69; 13.15]	[-2.21; 2.16]	[-1.89; 1.07]
R other last fixation	0.11	6.35	0.51	1.11
	[-1.41; 1.64]	[-1.59; 14.29]	[-1.74; 2.76]	[-0.44; 2.67]
P you first fixation	0.25	1.54	2.15	3.72
	[-1.46; 1.97]	[-5.38; 8.45]	[-0.84; 5.15]	[-0.64; 8.08]
P other first fixation	-9.24	3.22	-11.35	4.28
	[-231.28; 212.80]	[-3.55; 10.00]	[-44.74; 22.04]	[-0.10; 8.67]
P you second fixation	1.09**	-0.08	1.02	0.81
	[0.26; 1.92]	[-1.07; 0.92]	[-0.27; 2.30]	[-0.58; 2.20]
P other second fixation	1.24*	0.89	1.33	1.38
	[0.07; 2.40]	[-0.20; 1.98]	[-0.58; 3.25]	[-0.15; 2.91]
P you last fixation	-2.14**	2.46	-1.20	-2.20**
	[-3.64; -0.64]	[-5.53; 10.45]	[-3.44; 1.03]	[-3.79; -0.60]
P other last fixation	-1.78*	3.91	-1.17	-1.04
	[-3.36; -0.19]	[-4.09; 11.91]	[-3.55; 1.21]	[-2.62; 0.55]
S you first fixation	1.72	2.42	3.10*	3.75
	[-0.03; 3.47]	[-4.48; 9.31]	[0.05; 6.15]	[-0.59; 8.10]
S other first fixation	-23.60	2.00	-2.09	4.66*
	[-238.26; 191.06]	[-4.89; 8.88]	[-16.35; 12.16]	[0.24; 9.08]
S you second fixation	1.81***	1.05	1.71*	1.02
	[0.86; 2.76]	[-0.10; 2.20]	[0.37; 3.06]	[-0.49; 2.52]

	(1)<br>**CC up left**	(2)<br>**CC up right**	(3)<br>**CC down left**	(4)<br>**CC down right**
S other second fixation	-1.91	0.49	1.68	1.24
	[-5.39; 1.58]	[-0.85; 1.83]	[-0.43; 3.79]	[-0.31; 2.80]
S you last fixation	-0.68	3.92	0.06	-1.42
	[-2.26; 0.90]	[-3.98; 11.82]	[-2.18; 2.30]	[-3.07; 0.23]
S other last fixation	-0.57	5.13	-1.10	-1.15
	[-2.20; 1.07]	[-2.84; 13.10]	[-3.64; 1.43]	[-2.88; 0.57]
AIC	2900.10	2957.35	3166.69	3140.52
BIC	5122.59	5177.42	5386.60	5363.17
Log Likelihood	-1100.05	-1128.67	-1233.34	-1220.26
Num. obs.	4230	4201	4199	4232
Num. groups: subj_nb	88	88	88	88

***P<0.001, **P<0.01, *P<0.05. 95% confidence interval in parentheses. The significant coefficients are shown in bold. AIC, Akaike information criterion; BIC, Bayesian information criterion.

Supplementary table 10. Mediation analysis: fixed effects coefficient estimates using mixed effects linear models treating participants as random effects. Corresponds to Supplementary Figure 9.

	Mediator (Cooperation row total fixation duration)	Outcome (Cooperation choice)
(Intercept)	0.49*	-4.01***
	[0.44; 0.54]	[-4.83; -3.18]
Position columns (C right)	0.01	-0.04
	[-0.00; 0.01]	[-0.20; 0.12]
Position rows (C down)	-0.10***	0.53***
	[-0.13; -0.07]	[0.33; 0.73]
Trial number	-0.01***	-0.29***
	[-0.01; -0.00]	[-0.45; -0.13]
Cooperation row total fixation duration		2.47***
		[1.86; 3.08]
AIC	-16659.06	10040.38
BIC	-16543.07	10195.03
Log Likelihood	8344.53	-5000.19
Num. obs.	16862	16862
Num. groups	88	88

***P<0.001, **P<0.01, *P<0.05. 95% confidence interval in parentheses. The significant coefficients are shown in bold. AIC, Akaike information criterion; BIC, Bayesian information criterion.

SUPPLEMENTARY FIGURES

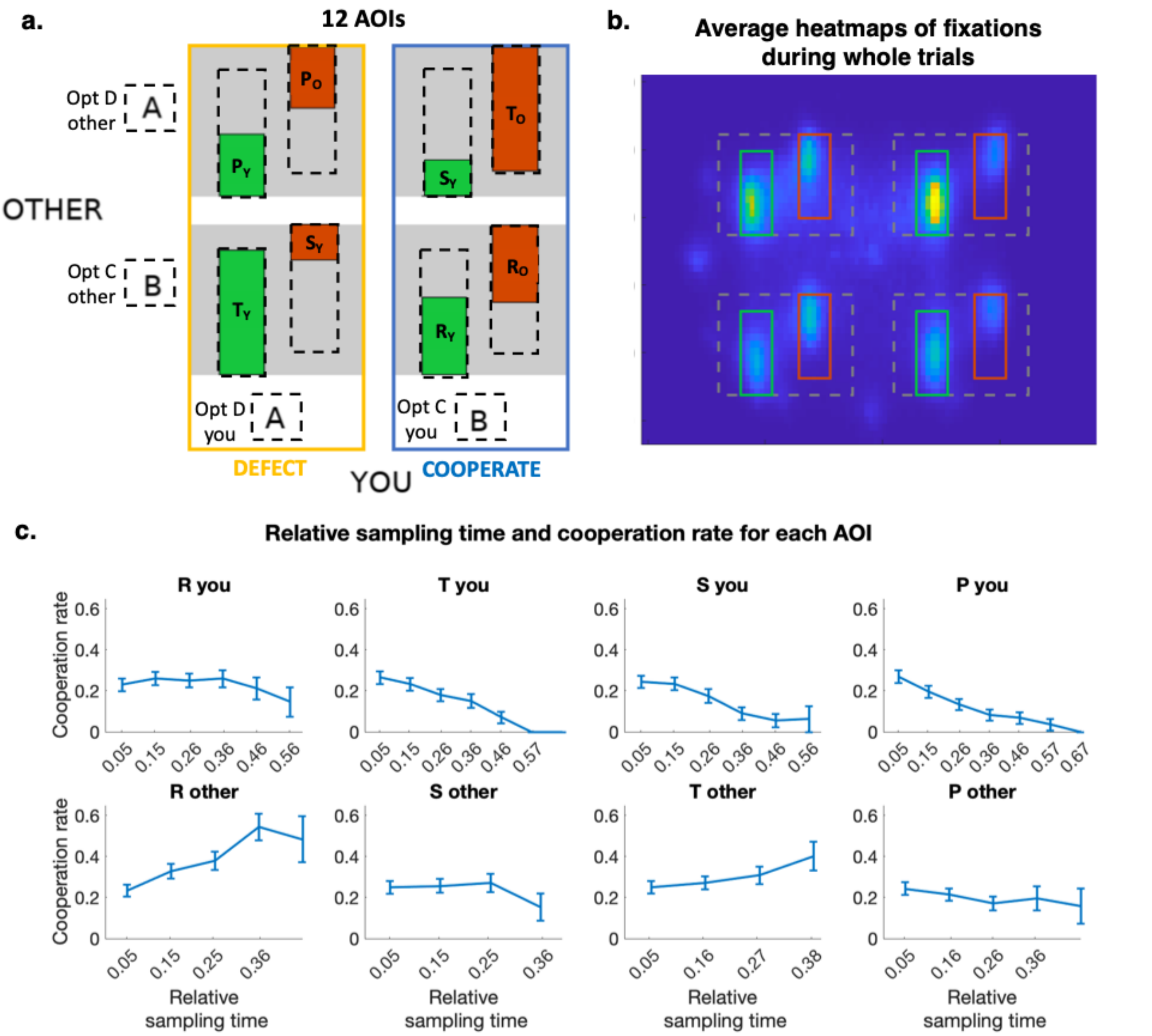

Supplementary Figure 1. Eye-tracking metrics: 12 AOIs, frequency and duration of fixations. a. *Twelve AOIs used for the eye-tracking analysis. Each AOI is represented using dashed lines. Eight AOIs correspond to the four possible payoffs (Temptation T, Reward R, Punishment P and Suckers S) for the two participants (main player, labelled YOU in green, oponnent, labelled OTHER in orange). Four AOIs correspond to the options labels (A and B) for each player.* ***b.*** *Average heatmap of eye positions during a trial (averaged across trials and participants). The x and y positions of the eye were measured, the frequency of each position is displayed, with bright (yellow) colors representing frequent positions and darker (blue) colors representing locations never gazed at. The rectangles represent the payoff AOIs for the main player (green) vs the oponnent (orange).* ***c.*** *Relative time spent sampling the 8 AOIs (time spent looking at each AOI divided by trial duration) and cooperation rate on the corresponding trial. The relative time spent was separated into bins with a 10% step size. The cooperation rate was computed across all trials within each bin (across all participants), the mean and the s.e.m of the cooperation rate for each bin are displayed. n = 88 participants.*

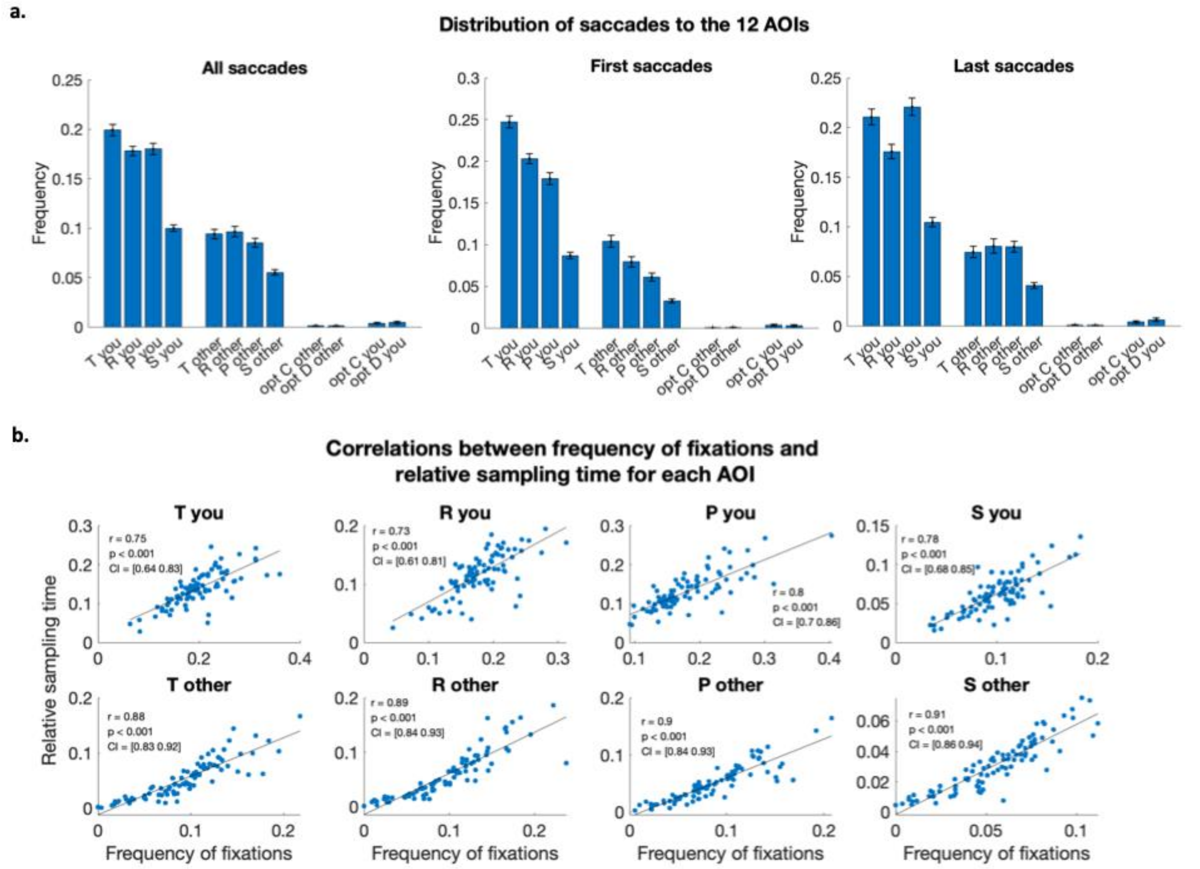

Supplementary Figure 2. Eye tracking metrics, frequency of sampling each AOI: a. *Distribution of saccades being to each of the 12 AOIs, averaged across participants. The bars represent the average across all participants, and the error bars are the standard errors of the mean. The left panel represents the overall distribution, across all fixations, the middle panel the first fixation, and the right panel the last fixation only. The four AOIs containing options labels were almost never sampled and therefore excluded from the rest of the analysis.* ***b.*** *Correlations between two metrics representing the relative importance of each AOI during a trial: the frequency of fixations to the AOI, and the relative time spent sampling the AOI during a trial (time spent looking at the AOI divided by trial duration). Each dot represents a participant and the line regressions between the metrics. The two metrics are highly correlated, we therefore restricted the rest of our analysis to the relative sampling time. All panels: n = 88 participants.*

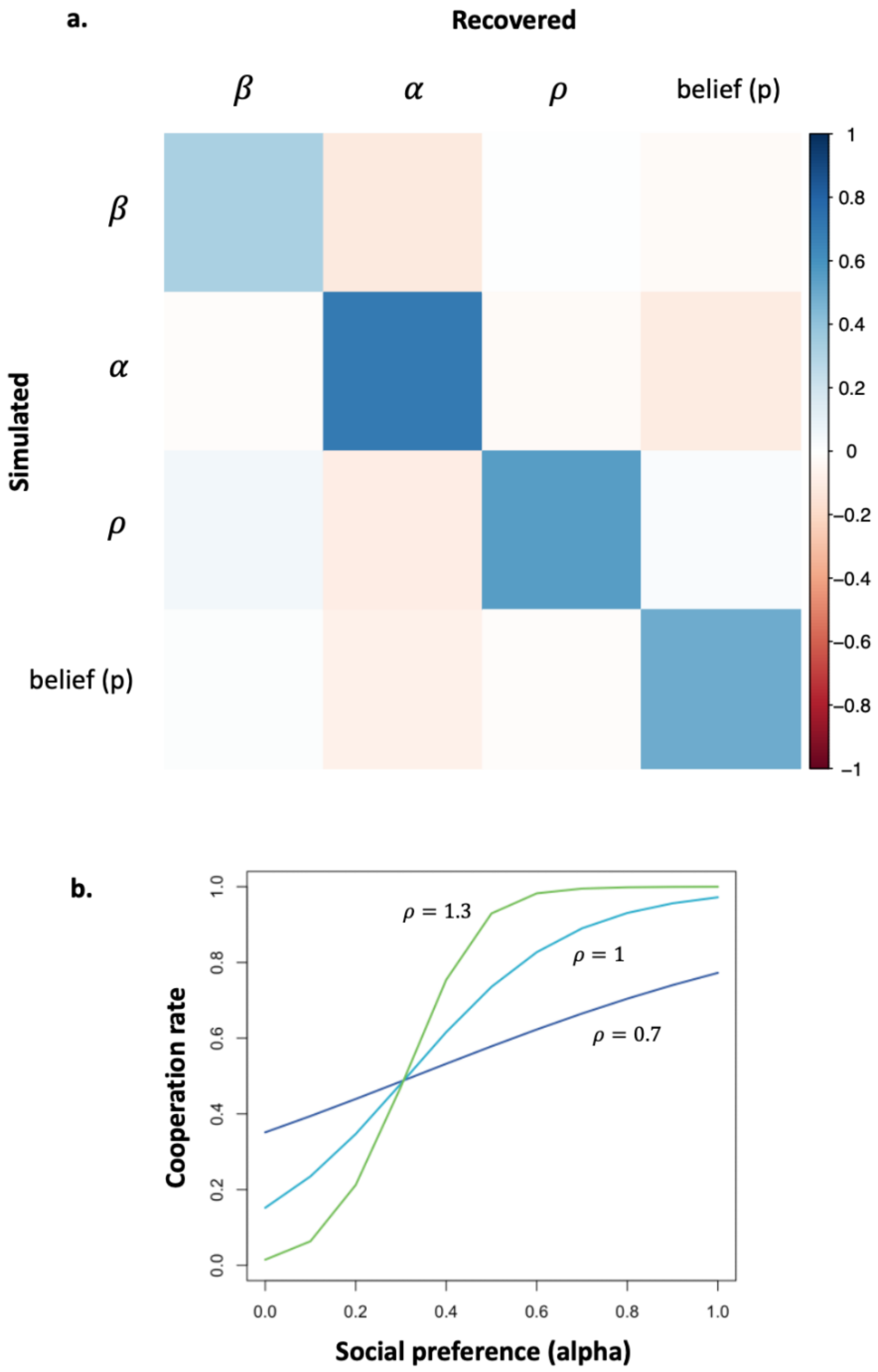

Supplementary Figure 3. a. Parameter recovery for the winning model. *We simulated 1000 participants with parameters randomly drawn from the intervals beta = [0, 1.3], alpha = [0, 1], rho = [0, 1.3], p = [0, 1] (these intervals were chosen to ensure reasonable changes in behavior in the task and model identifiability given our optimization procedure). The matrix shows Pearson correlation between the recovered and simulated values.* ***b. Example simulation of cooperation rates under different parameters.*** *The simulation uses the main model with p = 0.5, beta = 0.2, and variation in alpha from 0 to 1 and the risk parameter rho at values 0.7, 1, and 1.3. Note that under different levels of social preference (alpha) cooperation rates can either increase or decrease with the degree of risk aversion.*

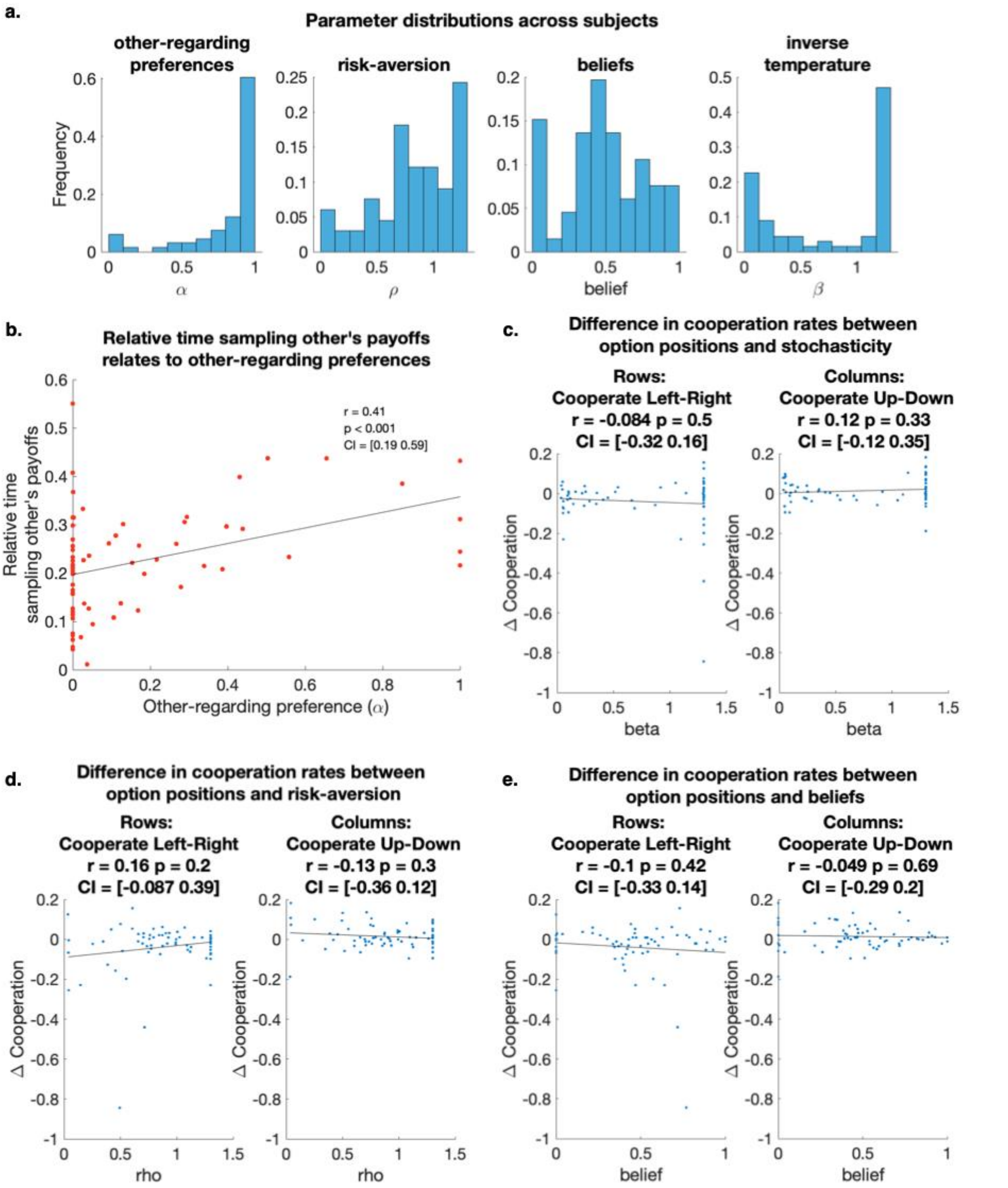

Supplementary Figure 4. Other-regarding preferences are linked to the sampling of others' payoffs, bottom-up attentional effects on cooperation are independent of individual preferences. a. *Distribution of the four parameters of the winning model across the 66 participants who cooperated or defected on at least one trial.* ***b.*** *Relation between the relative time spent sampling any payoff of other (T_O, R_O, P_O, S_O) and fitted other-regarding preferences. Each dot represents a participant, the line represent a fitted regression.* ***c-e.*** *Relations between the effects of row and column positions on cooperation (difference in cooperation for the positions) and fitted model parameters. Each dot represents a participant, the lines represent fitted regressions. All panels: n = 66 participants.*

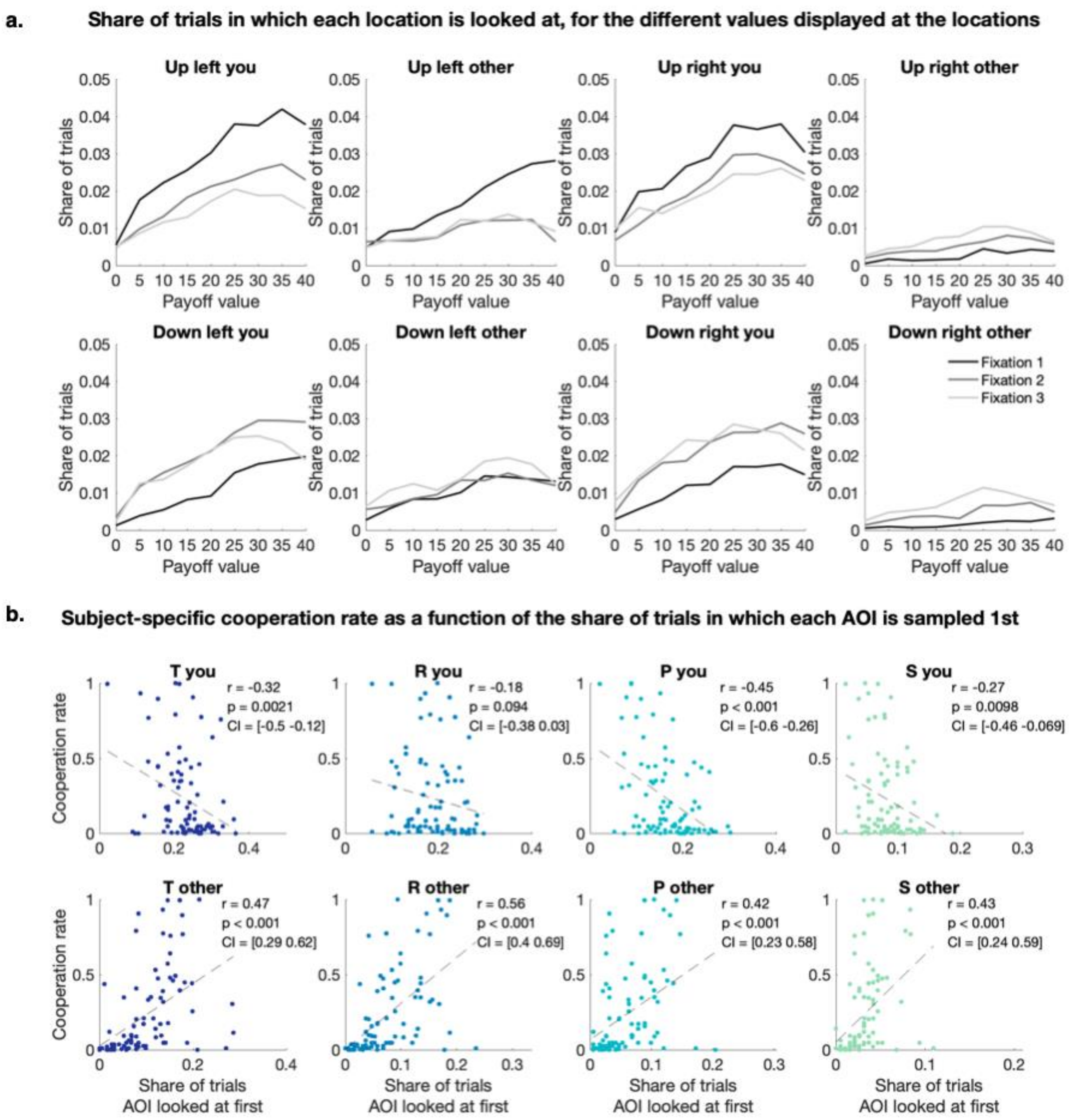

Supplementary Figure 5. Screen locations and payoff values influence sampling patterns, which relate to cooperation. a. *Effects of payoff values and fixation number (in each gaze sequence) on the share of fixations to different screen locations, across all trials and participants. The upper row is looked at most often during the 1st fixation, and when the values represented at these locations are high. The lower row is looked at more often during fixation 2 and 3 compared to the first fixations, and when the values represented at these locations are high.* ***b.*** *Proportion of trials in which participants sampled each AOI during the first fixation for each participant, and average cooperation. Each dot represents a participant, and the lines and statistics represent fitted regressions and correlation coefficients between the share of trials starting with each AOI and the cooperation rate. Participants starting trials mostly looking at their payoffs have a low cooperation rate, while participants starting with the payoffs of the other have a high cooperation rate. All panels: n = 88 participants.*

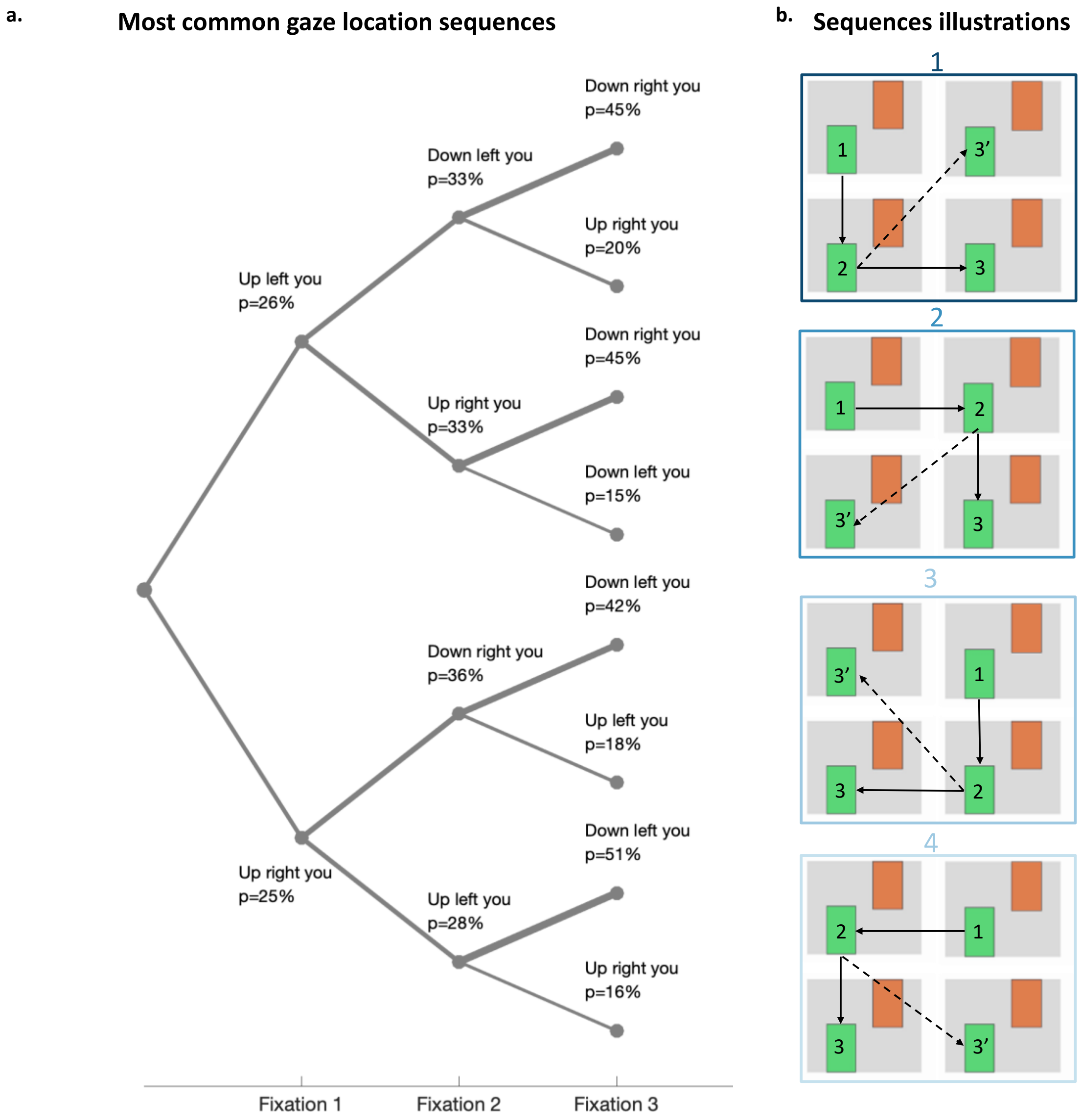

Supplementary Figure 6. Most common gaze location sequences. a. *Transition tree of the most frequent sequences. On each fixation, the two most frequent saccades are displayed, as well as the proportion of trials for which the transition to this screen location occurs (conditional on the previous fixation, displayed as a percentage).* ***b.*** *illustration of the most common sequences: the numbers correspond to the fixations, and arrows show the most frequent transitions. Dotted arrows show the second most frequent transition from fixation 2 to 3. N = 88 participants.*

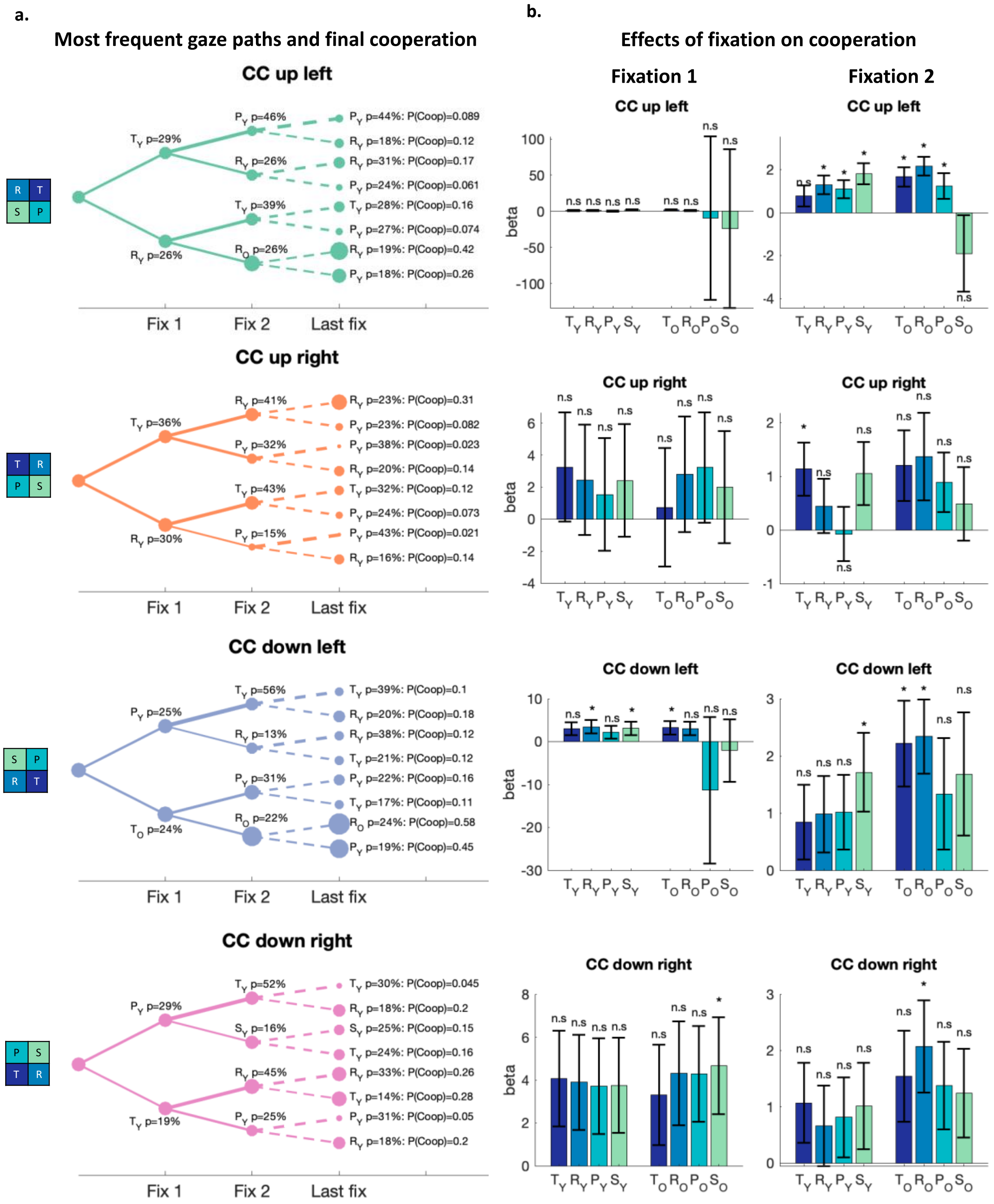

Supplementary Figure 7. Most frequent gaze sequence in the four positions, and effects of different fixations on cooperation. *The most common gaze sequence for the four option positions, cooperation rates, and probabilities of transitioning to each frequent AOI. On each fixation, the two most frequent saccades are displayed, as well as the proportion of trials for which the transition occurs (conditional on the previous fixation, displayed as a percentage), and the average cooperation rate across all trials of the sequence (displayed as different-sized bullets). The dotted lines correspond to multiple sequences leading to the most frequent last fixations. The final P(Coop) indicates the cooperation rate across trials following each gaze sequence.* ***b.*** *Fixed effects coefficients' estimates of dummies representing fixations of different AOI at different time points regressions on Cooperation using mixed-effects logistic models. A different model is fitted to each option positions and includes a dummy for fixation 1 and 2. The bar represent the beta estimates and error bars are the standard errors estimated by the model. Corresponds to Supplementary Table 9. All panels: n = 88 participants.*

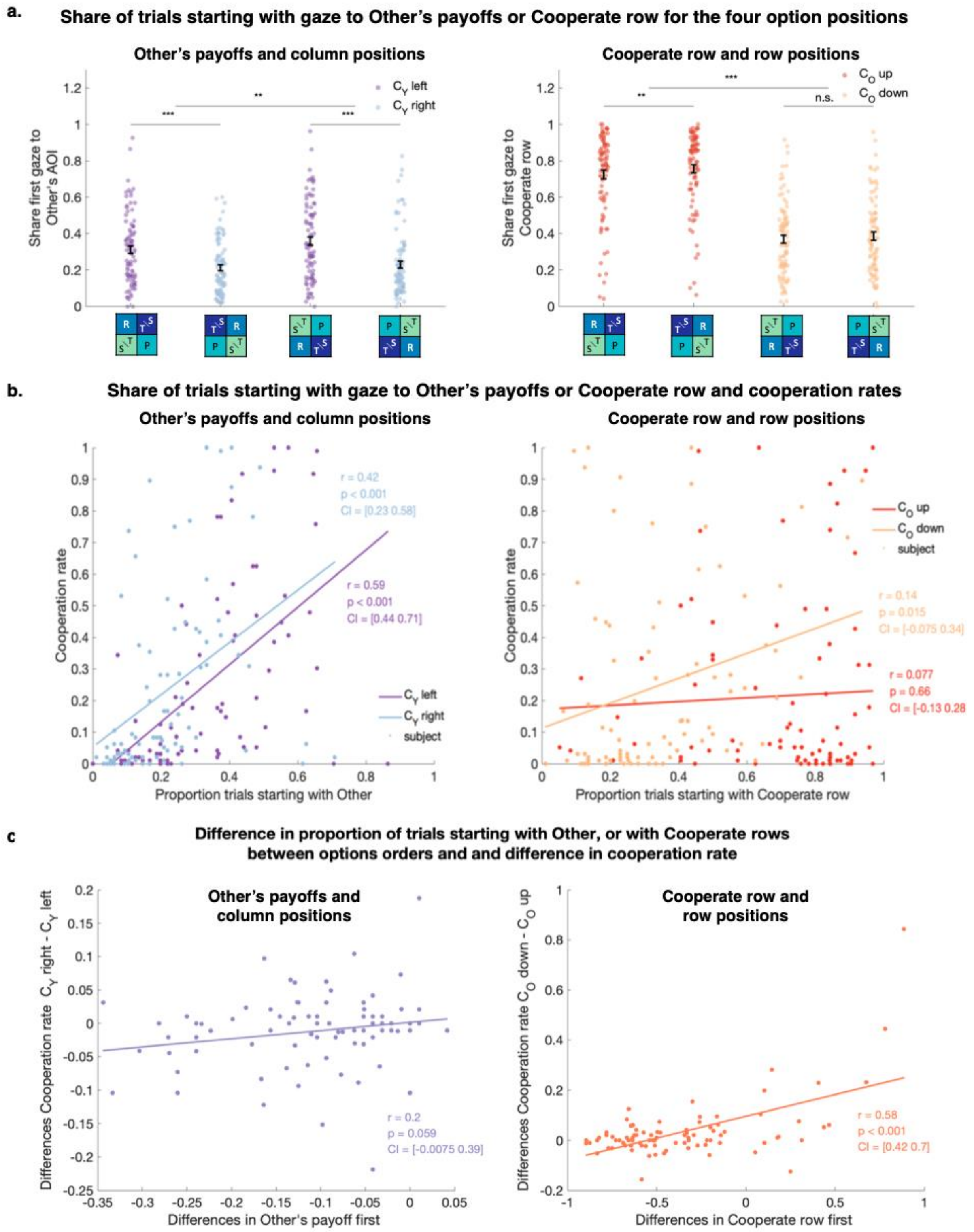

Supplementary Figure 8. Effects of first fixations to other or cooperate rows on cooperation and interactions with option positions. a. *Share of trials starting with AOIs for the other (left: other vs you) or AOI corresponding to a cooperation of the other player (right: row cooperate vs defect) for different column and row positions. The proportions are computed for each participant for each* participant (represented with a dot per participant)*, the* error*bars represent the average across* participants and *s.e.m. Significance stars represent a two-tailed paired t-test:* $p < 0.00033$ (***), $p < 0.0033$ (**), $p < 0.017$ *(*), or non-significant (n.s.), using a Bonferroni correction. In the left panel, the colors represent the column positions: Cooperate left vs Defect left, and show that participants start more often looking at the other's payoffs when their own cooperation option is placed on the left. In the right panel, the colors represent the row positions: Cooperate up vs Defect up, and show that participants start looking at the cooperate row more often when the cooperate option for the other is placed on top.* ***b.*** *Correlations between the share of trials starting with other (left panel) or with the cooperate row (right panel) and the cooperation rate of each participant, for the different positions of the columns (left panel) or rows (right panel).*

Participants who start looking at the other's options more frequently have a higher cooperation rate. Participants who start looking at the cooperate row more frequently have a higher cooperation rate, but only when this row is placed in the bottom. ***c.*** *Differences between the two column positions (left panel) or row positions (right panel) in proportions of trials with first fixation to the other (left panel) or to the cooperate row (right panel) and differences in cooperation rate. The increase in cooperation rate for each participant when the cooperation is placed at the bottom is proportional to the difference in the proportion of trials in which these participants start with the Cooperate row. All panels: n = 88 participants.*

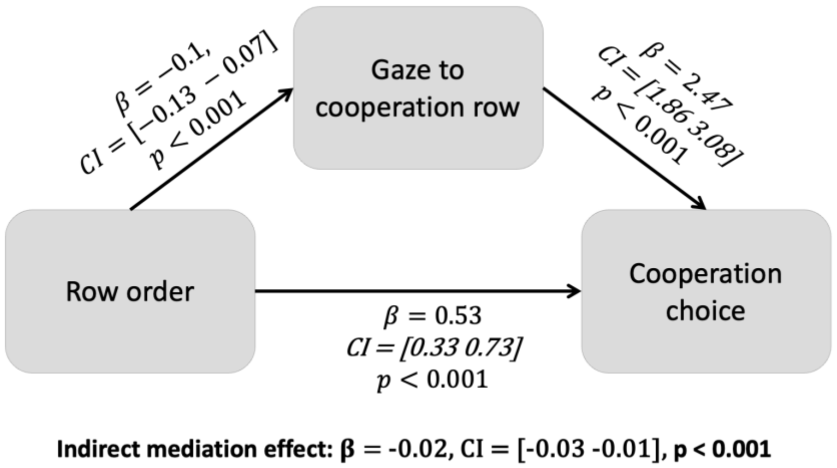

Supplementary Figure 9. *Mediation analysis results using individual trial choice (= 1 if cooperate) as the dependent variable, the trial-level row order as the independent variable, and relative gaze to the cooperation row as a mediator. All regressions are mixed effects models, including trial number as a control variable and participant-level random effects (see Supplementary Table 10). N = 88 participants.*
