# Supplementary material for: Manipulating attention facilitates cooperation
Source: Commun Psychol. 2025 Mar 17;3:39. doi: 10.1038/s44271-025-00206-9 (PMC11913732; doi:10.1038/s44271-025-00206-9)
Supplement: Supplementary file 3 — Reporting Summary [file 44271_2025_206_MOESM3_ESM.pdf]

nature portfolio

Corresponding author(s): Arkady Konovalov

Last updated by author(s): Sep 3, 2024

Reporting Summary

Nature Portfolio wishes to improve the reproducibility of the work that we publish. This form provides structure for consistency and transparency in reporting. For further information on Nature Portfolio policies, see our Editorial Policies and the Editorial Policy Checklist.

Statistics

For all statistical analyses, confirm that the following items are present in the figure legend, table legend, main text, or Methods section.

n/a	Confirmed	
☐	☒	The exact sample size (*n*) for each experimental group/condition, given as a discrete number and unit of measurement
☐	☒	A statement on whether measurements were taken from distinct samples or whether the same sample was measured repeatedly
☐	☒	The statistical test(s) used AND whether they are one- or two-sided<br>*Only common tests should be described solely by name; describe more complex techniques in the Methods section.*
☐	☒	A description of all covariates tested
☐	☒	A description of any assumptions or corrections, such as tests of normality and adjustment for multiple comparisons
☐	☒	A full description of the statistical parameters including central tendency (e.g. means) or other basic estimates (e.g. regression coefficient) AND variation (e.g. standard deviation) or associated estimates of uncertainty (e.g. confidence intervals)
☐	☒	For null hypothesis testing, the test statistic (e.g. *F*, *t*, *r*) with confidence intervals, effect sizes, degrees of freedom and *P* value noted<br>*Give P values as exact values whenever suitable.*
☒	☐	For Bayesian analysis, information on the choice of priors and Markov chain Monte Carlo settings
☒	☐	For hierarchical and complex designs, identification of the appropriate level for tests and full reporting of outcomes
☐	☒	Estimates of effect sizes (e.g. Cohen's *d*, Pearson's *r*), indicating how they were calculated

Our web collection on statistics for biologists contains articles on many of the points above.

Software and code

Policy information about availability of computer code

Data collection	MATLAB 2020a, Psychtoolbox 3, EyeLink 1000 Plus eye tracker system (SR Research Ltd.)
Data analysis	R 4.2.2, R packages lme4 1.1.18.1, lmerTest 3.0.1<br>Matlab 2020a<br>custom code available https://osf.io/z5pb7/

For manuscripts utilizing custom algorithms or software that are central to the research but not yet described in published literature, software must be made available to editors and reviewers. We strongly encourage code deposition in a community repository (e.g. GitHub). See the Nature Portfolio guidelines for submitting code & software for further information.

Data

Policy information about availability of data

All manuscripts must include a data availability statement. This statement should provide the following information, where applicable:

- Accession codes, unique identifiers, or web links for publicly available datasets
- A description of any restrictions on data availability
- For clinical datasets or third party data, please ensure that the statement adheres to our policy

The datasets generated during and analyzed during the current study are available in the OSF repository: https://osf.io/z5pb7/

Human research participants

Policy information about studies involving human research participants and Sex and Gender in Research.

Reporting on sex and gender	We collected self-reported sex, out of 88 subjects 48 self-reported as females and 40 as males. We did not have any a-priori hypotheses about sex or gender. We found no significant effect of gender on cooperation rates.
Population characteristics	This sample includes primarily university students (age 18 to 35 years old, mean: 24.6, std: 3.1)
Recruitment	Participants were recruited from the subject pool of the Department of Economics at the University of Zurich. The subjects self-select to the study when receiving an invitation email. We excluded subjects who had already taken part in similar economic exchange experiments (dictator games, trust games, prisoners dilemmas) as well as economics students.
Ethics oversight	Ethics committee of the Canton of Zurich (Kantonale Ethikkommission)

Note that full information on the approval of the study protocol must also be provided in the manuscript.

Field-specific reporting

Please select the one below that is the best fit for your research. If you are not sure, read the appropriate sections before making your selection.

☐ Life sciences ☒ Behavioural & social sciences ☐ Ecological, evolutionary & environmental sciences

For a reference copy of the document with all sections, see nature.com/documents/nr-reporting-summary-flat.pdf

Behavioural & social sciences study design

All studies must disclose on these points even when the disclosure is negative.

Study description	We collected quantitative data on cooperative decisions across situations with varying quantitative decision outcomes. We collected eye-tracking data (position of the pupil on the screen) while subjects were taking their decisions.
Research sample	88 subjects were recruited from the subject pool of the Department of Economics at the University of Zurich (48 females, 40 males, mean age 24.6). This sample is not representative of the general population, but is composed of a variety of individuals, mostly students, of different nationalities. Such sample allows us to study basic processes underlying individuals' social cognition, rather than characterize the behavior of the general population.
Sampling strategy	We determined the target sample size (N = 84) needed to estimate a significant correlation (significance level of 0.05, power 80%) between an individual’s eye tracking measures and behavioral measures such as cooperation rate, assuming a weak relationship with a Pearson correlation coefficient of 0.3.
Data collection	The data was collected at the Laboratory for Social and Neural System research of the University of Zurich, using a standard computer and an eye-tracker EyeLink 1000 Plus eye tracker system (SR Research Ltd.).
Timing	The data was collected between September and December 2021.
Data exclusions	We did not exclude data from any participants.
Non-participation	No participants dropped out during the experiment.
Randomization	There were no different experimental groups.

Reporting for specific materials, systems and methods

We require information from authors about some types of materials, experimental systems and methods used in many studies. Here, indicate whether each material, system or method listed is relevant to your study. If you are not sure if a list item applies to your research, read the appropriate section before selecting a response.

Materials & experimental systems

n/a	Involved in the study
☒	☐ Antibodies
☒	☐ Eukaryotic cell lines
☒	☐ Palaeontology and archaeology
☒	☐ Animals and other organisms
☒	☐ Clinical data
☒	☐ Dual use research of concern

Methods

n/a	Involved in the study
☒	☐ ChIP-seq
☒	☐ Flow cytometry
☒	☐ MRI-based neuroimaging
